# Neutrophil plasticity enables the development of pathological microenvironments: implications for cystic fibrosis airway disease

**DOI:** 10.1186/s40348-016-0066-2

**Published:** 2016-12-05

**Authors:** Camilla Margaroli, Rabindra Tirouvanziam

**Affiliations:** 1 Department of Pediatrics, Emory University School of Medicine, Atlanta, GA 30322 USA; 2 Center for CF and Airways Disease Research, Children’s Healthcare of Atlanta, Atlanta, GA 30322 USA; 3 Emory + Children’s Center, 2015 Uppergate Dr NE, Rm 344, Atlanta, GA 30322-1014 USA

**Keywords:** Amino acids, Exocytosis, Glucose, Inflammation, Immunometabolism, Reprogramming

## Abstract

**Introduction:**

The pathological course of several chronic inflammatory diseases, including cystic fibrosis, chronic obstructive pulmonary disease, and rheumatoid arthritis, features an aberrant innate immune response dominated by neutrophils. In cystic fibrosis, neutrophil burden and activity of neutrophil elastase in the extracellular fluid have been identified as strong predictors of lung disease severity.

**Review:**

Although neutrophils are generally considered to be rigid, pre-programmed effector leukocytes, recent studies suggest extensive plasticity in how neutrophil functions unfold upon recruitment to peripheral tissues, and how they choose their ultimate fate. Indeed, upon migration to cystic fibrosis airways, neutrophils display dysregulated lifespan, metabolic activation, and altered effector and regulatory functions, consistent with profound adaptation and phenotypic reprogramming. Licensed by signals present in cystic fibrosis airway microenvironment to survive and develop these novel functions, neutrophils orchestrate, in partnership with the epithelium and with the resident microbiota, the evolution of a pathological microenvironment. This microenvironment is defined by altered proteolytic, redox, and metabolic balance and the presence of stable luminal structures in which neutrophils and microbes coexist.

**Conclusions:**

The elucidation of molecular mechanisms driving neutrophil plasticity in vivo will open new treatment opportunities designed to modulate, rather than block, the crucial adaptive functions fulfilled by neutrophils. This review aims to outline emerging mechanisms of neutrophil plasticity and their participation in the building of pathological microenvironments in the context of cystic fibrosis and other diseases with similar features.

## Introduction

Neutrophils constitute the first line of defense against infection in most organisms. It is estimated that the human body produces 10^9^ neutrophils/kg/day, making them the most abundant leukocytes in bone marrow (BM) and blood. Neutrophils play an important role in protective immunity, which explains the severe pathologies arising upon hereditary or acquired impairment of neutrophil number and function. Blood neutrophils are conventionally thought of as terminally differentiated cells with little license to adapt to conditions within tissues beyond their ability to kill pathogens intracellularly by phagocytosis, or extracellularly by degranulation or release of DNA-based neutrophil extracellular traps (or NETs) in a recently discovered process dubbed “NETosis”.

However, in the context of cystic fibrosis (CF) lung disease, neutrophils show complex properties, detailed below, that come in stark contrast with the rigid pre-programmed phenotype generally expected of them and instead emphasize their inherent plasticity. CF is a hereditary, recessive disease that predominately impacts individuals of European ancestry. According to the World Health Organization, its incidence varies between 1 in 2000 and 1 in 3500 newborns worldwide. The gene mutated in CF patients encodes the CF transmembrane conductance regulator (CFTR), an ATP-binding cassette family member that regulates the movement of anions, such as chloride, bicarbonate, thiocyanate, and glutathione (GSH), across the plasma membrane [1, 2]. So far, more than 1800 disease-causing mutations have been identified among CF patients, with the F508Del mutation being the most frequent (~70% of mutated alleles) [3, 4]. Digestive enzyme supplements have noticeably increased CF patients’ lifespan and shifted the main cause of morbidity from nutrient malabsorption due to pancreatic failure to chronic lung disease [5].

Impaired mucociliary clearance, bacterial infection, and neutrophilic inflammation are all hallmarks of CF lung disease [6, 7]. Among those, neutrophil burden and extracellular activity of the protease neutrophil elastase (NE) in CF airway fluid correlate best with disease progression in CF patients, from infancy to adulthood [8]. The role of neutrophil inflammation in CF pathophysiology has been exhaustively reviewed elsewhere [9–11]. Recent reviews detail the putative role of other immune cells, such as macrophages, in CF lung disease [12–14]. In the present review, our goal is to direct the attention of the reader to the phenotypic reprogramming process that neutrophils undergo in the context of CF lung disease, and explore potential mechanisms and treatment opportunities afforded by this newly discovered process. Importantly, this new view of neutrophils, which we illustrate in the context of CF, echoes recent findings made in the context of acute infection and sepsis, as well as other chronic inflammatory diseases such as chronic obstructive pulmonary disease (COPD), rheumatoid arthritis (RA), and systemic lupus erythematosus, as well as cancer, where neutrophils also display new, complex phenotypes and effector functions [15].

## Review

### Neutrophil plasticity in CF lung disease: emergent mechanisms

#### Lifespan and aging

Pulse-chase experiments were conducted recently to measure the lifespan of human neutrophils in blood. Models accounting for the loss of the deuterium label led to estimates of a few hours to up to 5 days [16, 17]. Although their exact lifespan is debated, it is of general consensus that neutrophils leave the BM with a default pro-apoptotic program that can be inhibited by stimuli received upon migration to peripheral tissues [18, 19]. In CF, there is no experimental data on the precise lifespan of neutrophils in the lung, and this subject remains debated. On the one hand, the hostile environment of the CF lung, and notably the presence of bacterial toxins, could induce rapid necrosis of incoming neutrophils [20–22]. On the other hand, neutrophil lifespan may be extended by several factors, such as pro-survival signals from neutrophils, as well as exogenous drugs, and epithelial and microbial inflammatory mediators and metabolites. For example, Sutanto et al. [23] showed that primary epithelial cells from CF infants not only secrete higher levels of inflammatory mediators compared to their healthy counterparts at baseline but also display an increased production of interleukin-8 (IL-8) in response to human rhinovirus infection. In addition to being a strong chemoattractant [24], IL-8 can delay neutrophil apoptosis [25]. Thus, the CF airway epithelium may contribute to a higher lifespan of neutrophils recruited to the lumen.

To achieve a balanced number of neutrophils in blood, the high daily rate of release of mature young neutrophils into the bloodstream is compensated by the clearance of senescent neutrophils from it. Circadian rhythm is a major factor influencing hematopoiesis in general and neutrophil turnover in particular [26–28]. From a phenotypic standpoint, developing neutrophils in the bone marrow (BM) express the chemokine receptor CXCR4, which acts as a retention signal by binding to its cognate ligand CXCL12 on stromal cells. The release of mature neutrophils into the circulation coincides with the downregulation of CXCR4 expression, and concomitant increase in expression of CXCR2, a receptor for IL-8. However, senescent neutrophils increase CXCR4 expression again [17], which is thought to lead to their return to the BM where they are cleared by resident macrophages. In addition to its role in mediating cell retention in the BM, CXCR4 signaling has been proposed as a direct regulator of neutrophil lifespan. In mice, CXCR4 expression is increased in neutrophils after migration to the lungs and correlates with increased lifespan [29]. In patients with COPD, neutrophils are present in large numbers within the bronchoalveolar lavage fluid (BALF) [30] and express higher levels of CXCR4 compared to control subjects [31]. Similarly, neutrophils isolated from the sputum of CF patients showed increased surface expression of CXCR4 compared to blood neutrophils [32], and its ligand CXCL12 was detected in some CF sputum samples, suggesting a potential role of this pathway within the CF airway lumen.

In addition to the CXCR4/CXCL12 axis, new insights from the zebrafish model of neutrophil development show that signaling through the oxygen-dependent transcription factor hypoxia-inducible factor-1α (HIF-1α) can also significantly delay neutrophil apoptosis [33]. Since affected areas in CF lungs become highly hypoxic due to mucus impaction and fast oxygen consumption by activated neutrophils [34], it is tempting to speculate that HIF-1α signaling may be triggered in neutrophils present in this pathological microenvironment, affecting their lifespan. Consistent with this notion, significant HIF-1α signaling has been demonstrated in the βENaC mouse model of CF lung disease, inducing substantial pro-inflammatory signaling within the epithelium that results in neutrophilic inflammation [35].

Interestingly, it has been suggested that neutrophils in CF patients have an intrinsic increase in lifespan due to the mutation of the *CFTR* gene. Indeed, *ex vivo* experiments on blood neutrophils isolated from healthy controls and CF patients with the F508Del mutation showed delayed apoptosis in the latter [36, 37]. However, these data do not imply increased lifespan in vivo. Also, since ongoing treatments can significantly impact neutrophil behavior [38], it is likely that drugs administered to CF patients from whom neutrophils are collected can alter the lifespan of these cells *ex vivo*. Another interesting factor to consider when reflecting on potential influences exerted onto neutrophil lifespan is that of the resident microorganisms. Indeed, it has been demonstrated that neutrophil biogenesis and aging in mice is controlled, in part, by the gut microbiome [39, 40]. In patients with chronic infections, e.g., CF or COPD, it is likely that the lung microbiome could also play a role in shaping neutrophil lifespan [41], although this notion remains controversial [42, 43].

In the context of a normal immune response to an insult, increased neutrophil lifespan can be beneficial for the host, at least temporarily. However, if this response becomes dysregulated, it can constitute a double-edged sword [44]. To this day, many questions relative to the recruitment of neutrophils and their precise lifespan within the CF lung in vivo remain unanswered. A key difficulty resides in studying these mechanisms in vivo, and in untangling factors intrinsic to CF (compared to other diseases with similar neutrophilic inflammation, such as COPD), and those affected by exogenous drugs. Finally, since neutrophils, the airway epithelium, and microorganisms all contribute to CF lung disease, integrative approaches combining signals from all components of this pathological microenvironment are needed to yield better understanding of the mechanisms at play.

#### Overview of effector functions

In the course of inflammation, neutrophils recruited from blood cross into tissues and organize themselves in “swarms” to travel to the site of injury. Neutrophil migration responds to gradients of exogenous and autocrine/paracrine chemokines (e.g., IL-8), cytokines (e.g., tumor necrosis factor α), as well as bioactive lipids (e.g., leukotriene B_4_ (LTB_4_)) [45, 46]. Dynamic expression of specific receptors to these chemokines, cytokines, and bioactive lipids is critical to neutrophil migration. In addition, neutrophils express a plethora of pattern recognition receptors (PRRs) that allow them to sense and capture signals present in their surroundings in the form of pathogen-associated molecular patterns (PAMPs) or danger-associated molecular patterns (DAMPs) [47]. PAMPs (e.g., lipopolysaccharide from gram-negative bacteria) and DAMPs (e.g., extracellular advanced glycation endproducts or adenosine triphosphate) are present in damaged tissues and play a major role in influencing the functional fate of incoming neutrophils, notably the mobilization of intracellular granules.

Neutrophil granules are designated based on their content and order of production during BM development. Primary or azurophilic granules are formed at the early stages of neutrophil lineage formation in the BM (myeloblast to promyelocyte) and contain the potent proteases neutrophil elastase (NE) and cathepsin G, the chlorinating enzyme myeloperoxidase (MPO), and defensins. Secondary or specific granules arise at the later metamyelocyte stage and are characterized by the presence of lactoferrin, collagenase, carcinoembryonic antigen cell adhesion molecule family members CD66a and CD66b, and the antiprotease cystatin C. Tertiary or gelatinase granules appear at the band cell stage, right before the final segmented stage of neutrophil BM development, and enclose lysozyme and matrix metalloproteinase 9 (MMP9). Secretory vesicles are present only in mature neutrophils and are thought to be produced by endocytosis of surface expressed proteins, enabling their rapid redeployment at the surface upon activation, as exemplified by the upregulation of PRR surface expression upon priming in blood [48, 49]. Mobilization of secretory vesicles followed by tertiary and secondary granules appears to be a default activation path for neutrophils. By contrast, primary granules generally fuse either with the phagosome or the nucleus [50], the latter being part of the recently discovered NETotic fate of neutrophils. During NETosis, DNA is decondensed, released along with histones, and complexed with cationic primary granule proteins (chiefly NE and MPO), thus forming extracellular traps endowed with antimicrobial activities [51].

**Fig. 1:**
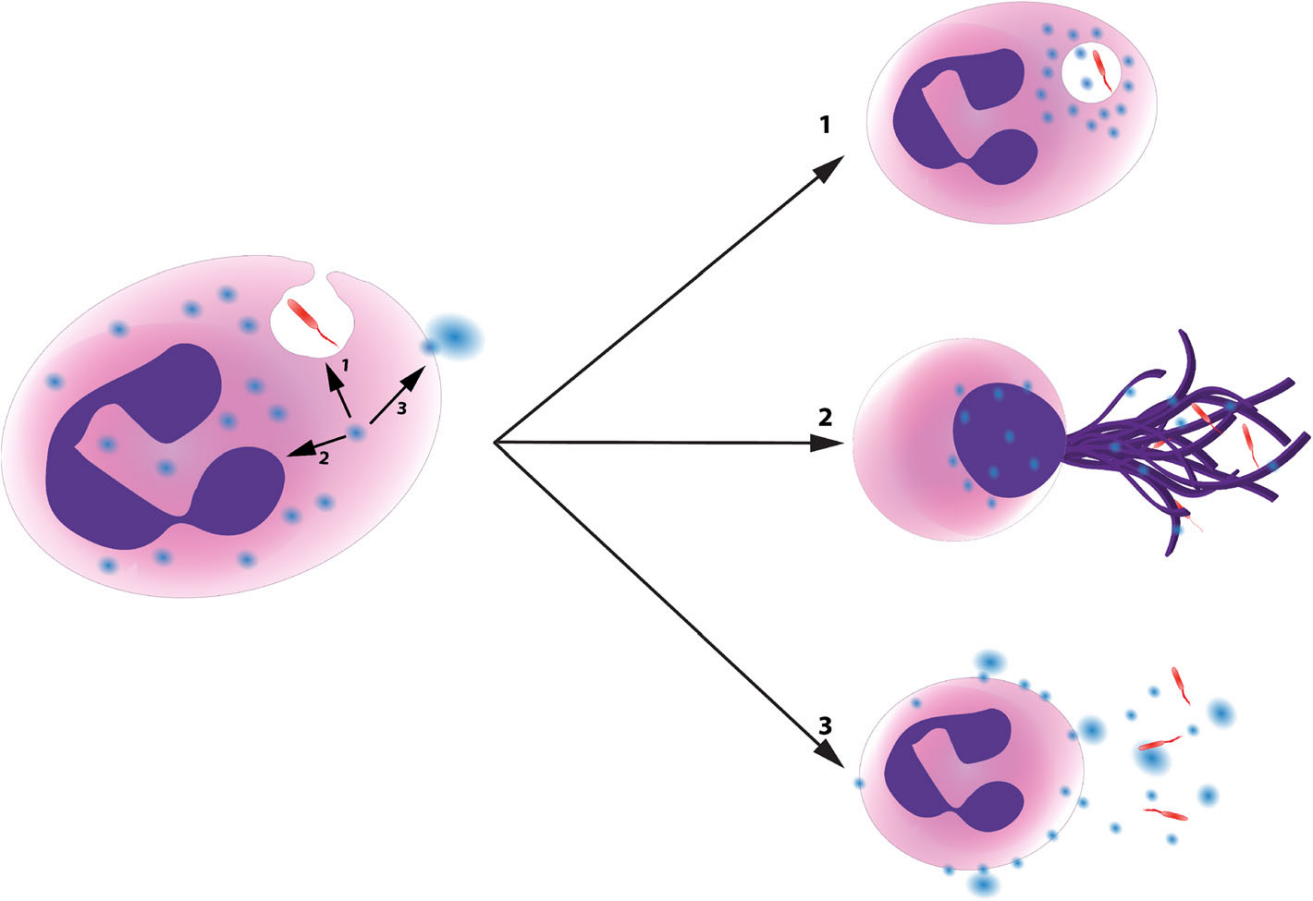
Primary granule mobilization and functional fates of human neutrophils. Recent studies have revealed the existence of multiple functional fates of neutrophils, which lead to different interactions with incoming bacteria (*in red*). Fusion of primary granules (*in blue*) to the phagosome drives neutrophils toward the classical phagocytic fate (*1*), while their mobilization to the nucleus drives them toward the extrusion of DNA-based extracellular traps in a process called “NETosis” (*2*). By contrast, primary granule fusion with the plasma membrane instead leads to hyperexocytosis and potential reprogramming (*3*), a third fate that further emphasizes the functional plasticity of human neutrophils

Until recently, it was believed that, due to the high self-harming potential of primary granule enzymes, the content of primary granules was rarely if ever discharged actively in the extracellular environment during the normal course of an inflammatory response. Thus, the massive amounts of NE and MPO present in the pathological milieu of CF and COPD airway fluid were thought to stem from the passive release of primary granules following neutrophil necrosis. However, the discovery of viable neutrophils in the CF lung lumen capable of active primary granule exocytosis has overturned this belief [52]. In these cells, mobilization of primary granules to the plasma membrane is not a passive outcome, but rather a finely orchestrated mechanism leading to a fate distinct from phagocytosis and NETosis (Fig. 1). The molecular mechanisms behind the differential primary granule mobilization to the phagosome (phagocytosis), nucleus (NETosis), or plasma membrane (third, and presumably distinct, fate), and whether each of the three described fates is exclusive of the others, are but a few examples of the current mysteries surrounding neutrophil biology that will have to be addressed in future research.

#### Focus on neutrophil elastase

A major effector of neutrophils with a critical role in CF is neutrophil elastase (NE), a serine protease composed of 218 amino acids. First discovered in 1968 by Janoff and Scherer [53] in the granular fraction of neutrophils, it took 15 more years for the sequence of NE to become known [54]. Upon primary granule release, the majority of NE remains bound to the plasma membrane [55, 56], which enables it to have its catalytic region facing the extracellular environment while concealing its regulatory region, thus making inhibitors less effective. The important pathophysiological role played by NE is highlighted by the fact that NE-knockout mice are highly susceptible to sepsis induced by gram-negative bacteria [57]. In humans, cyclic neutropenia, a genetic disease caused by mutations in the NE-coding ela2 gene, is associated with recurrent troughs in neutrophil production and heightened susceptibility to infections, suggesting a dual developmental and functional role for NE [58, 59].

In CF, increased presence of active NE in the airway fluid of pediatric and adult patients has been correlated with impaired structural integrity, worsening lung function, and decreased body mass index over time [60–63]. In a recent study, detectable NE activity in BALF of 3-month-old CF infants was the best predictor of future bronchiectasis development, with a likelihood seven times higher at 12 months and four times higher at the age of three than 3-month-old CF infants with no detectable NE in BALF activity [62]. Since BALF is highly diluted, due the way it is collected, it is possible that detection of free NE activity in CF infants happens only after constant NE release has overcome the secreted antiprotease shield present in the airways, thereby crossing a certain pathological threshold. Thus, more sensitive methods for extracellular NE detection are required in order to detect abnormal neutrophilic inflammation before it reaches a critical level and causes significant pathology.

Complementing early work by Owen and colleagues demonstrating NE activity in close vicinity to the plasma membrane of exocytosing neutrophils [55], additional work needs to be performed to determine the exact localization of NE in the extracellular environment within CF and COPD airways in vivo. Recently, Schulenburg et al. [64] designed a Förster resonance energy transfer probe specific for NE activity that could serve such a purpose. Potential applications of such probes have been extensively reviewed elsewhere [65]. Due to the wide range of proteins with NE cleavage sites that could potentially serve as NE substrates within pathological environments (see Table 1), it is hard to predict which of these proteins will be effectively proteolyzed in vivo. Among these proteins, one finds both immunological and non-immunological target proteins expressed by neutrophils, T cells, macrophages, and epithelial cells. Further adding to this complex picture, NE can be acquired by neighboring cells following its release by neutrophils [66]. This effectively extends the number of possible targets of NE-mediated cleavage to include intracellular proteins, which in turn affect signaling in neighboring cells (Table 2).

**Table 1:** Direct targets of NE-dependent regulation

Immunological targets	
Activated by cleavage	Inhibited by cleavage
Arginase-1 [106]	CD2/CD4/CD8 [107]
Chemerin [161]	CD14 [162]
IL-36 receptor antagonist [163]	CD16 [164]
IL-8 [84]	CD43 [165]
MMP-9 [67]	CCL3 [166]
PAR-1/PAR-2 [167, 168]	Complement factors [169–172]
Pro-IL-1β [173]	CXCL12 [174]
Transient receptor potential vanilloid 4 [67]	CXCR1 [82]
Tumor growth factor α [175]	IgA [70] and IgG [71, 72]
	IL-2 receptor [108]
	IL-6 [176, 177]
	IL-8 [86]
	PAR-3 [168]
	Progranulin [178, 179]
	TIMP-1/TIMP-2/TIMP-3 [180, 181]
Non-immunological targets	
Activated by cleavage	Inhibited by cleavage
α_2_β_3_ integrins [182]	Cadherins [183]
EGFR [184]	Elastin [185]
ENaC [186, 187]	Ferritin [188]
	Fibrin stabilizing factor XIII [189]
	Surfactant protein A [190]
	Surfactant protein D [191]
	Vascular endothelial growth factor [192]

*EGFR* epidermal growth factor receptor, *ENaC* epithelial sodium channel, *PAR* protease-activated receptor, *TIMP* tissue inhibitor of metalloproteinase

**Table 2:** Indirect targets of NE-dependent regulation and cognate signaling pathways

Regulatory target	Modulation by NE	Signaling pathway
β-defensin 2 [193]	Activation	Unknown
Cathepsin B [194]	Activation	TLR4/IRAK
CFTR [97]	Inhibition	Calpain
*P. aeruginosa* flagellin [81]	Inhibition	Unknown
IL-12 p40 [195]	Activation	PAR-2/EGFR/TLR4
IL-8 [83, 192, 196, 197]	Activation	TLRs/MyD88/IRAK/TRAF-6
MHC I [198]	Activation	Unknown
MMP-2 [194]	Activation	TLR4/IRAK
MUC5AC [199, 200]	Activation	EGFR

*EGFR* epidermal growth factor receptor, *ERK* extracellular-regulated kinase, *IRAK-1* IL-1 receptor associated kinase-1, *MHC I* major histocompatibility complex I, *PAR-2* protease-activated receptor-2, *TLR4* Toll-like receptor 4

A prototypical example highlighting the impact of unopposed NE activity in a pathological milieu is its ability to activate MMP9, another potent neutrophil protease. Upon concomitant release of primary and tertiary granules, NE can potentiate MMP9 through direct activatory cleavage and/or indirect degradative cleavage of its inhibitor tissue inhibitor of metalloprotease-1 (TIMP-1), leading to increased collagen degradation, tissue damage, and bronchiectasis in CF children [67, 68]. Likewise, surface phagocytic receptors CD14 and CD16 on neutrophils found in the lumen of CF patients’ lungs are inactivated by NE in autocrine and paracrine fashion [69]. Moreover, antibody-mediated bacterial killing is impaired not only on the receptor side but also on the opsonization potential of the antibody. As matter of fact, it has been shown that NE can cleave immunoglobulins A (IgA) [70] and G (IgG) [71, 72] near their hinge region. This leads to the formation of Fab and Fc fragments that are able to bind *separately* to the bacteria and receptors on target cells, thus losing the adaptor function of the antibody [73, 74]. In addition to NE, the CF opportunistic pathogen *Pseudomonas aeruginosa* also contributes its own elastase activity, which can also cleave IgG [75]. This dual inhibition exerted by NE on antibodies present in the CF airway lumen has implications for the design of vaccine strategies aiming to induce anti-bacterial responses in CF, suggesting that these may be severely limited by the high extracellular NE burden. Another example of effector function modulation by NE is the cleavage of the IL-8 receptor CXCR1, associated with impaired bacterial killing [76]. This may contribute to the infection by opportunistic bacteria such as *Staphylococcus aureus* and *P. aeruginosa*, which are also hallmarks of CF lung disease [77, 78]. Whether NE-mediated damage is a primary cause of these persistent infections or whether other elements in the CF lung environment also contribute to impaired clearance of these specific bacteria remains a matter of debate [79, 80].

Beyond the failed clearance of these bacteria, their continued adaptation to the CF environment, notably their switch to mucoid and biofilm resistance phenotypes, may also benefit from NE activity. Indeed, NE-mediated activity can repress flagellin transcription in *P. aeruginosa*, which facilitates biofilm formation [81]. Interestingly, NE-produced fragments of CXCR1 were identified as potential contributors to epithelial activation and release of IL-8 in a Toll-like receptor 2 (TLR2)-dependent manner [82], thus creating a pathological feedback loop of neutrophil recruitment, NE release, CXCR1 cleavage on epithelial cells, and further neutrophil recruitment. Additional contributions to this positive feedback loop come from the NE-mediated transcriptional upregulation of IL-8 via MyD88/IRAK/TRAF-6 [83] and direct NE-mediated processing of IL-8 in the extracellular milieu [84]. Indeed, IL-8 is produced as a 99-amino acid precursor protein which is proteolytically cleaved at its N-terminus before release [85]. Once in the extracellular milieu, IL-8 can be further processed by extracellular proteases, such as NE, leading to different bioactive forms that vary from 77 to 69 amino acids in length, with the 72-amino acid form being the most potent [84]. In vitro studies also suggest that IL-8 can be ultimately degraded in an NE-dependent manner over time [86] which could serve to balance out the induction of IL-8 production and its post-transcriptional activation by NE.

It is worth mentioning that although exocytosis of primary granule content can be considered a hallmark of CF lung disease, this process is not homogeneously expressed among all neutrophils found in the CF airway lumen. Indeed, Makam et al. [32] proposed a subset classification of airway neutrophils based on their surface phenotype, with neutrophils initially migrating into the lumen and expressing low CD63 (limited primary granule exocytosis) and high CD16 expression on their surface, followed by the acquisition of high surface CD63 expression (high primary granule exocytosis) and concomitant loss of surface CD16. This striking phenotypic and functional transition and its implications for CF pathogenesis are discussed in more details below.

#### Impact of CFTR on neutrophil function

In humans, the impact of endogenous CFTR in shaping neutrophil effector functions is unclear, due in part to limitations in research tools. First, animal models, such as CFTR knockout mice, ferrets, pigs, and rats, still have not allowed researchers in the field to adequately recapitulate the natural history of CF lung disease as seen in patients, and particularly the central role played by neutrophils. Second, the CFTR protein is generally expressed at low levels in cells, which, combined with the paucity of reliable anti-CFTR antibodies, has made it difficult to establish the presence of significant CFTR expression in human neutrophils and how it may impact their function [87–89].

To date, several lines of evidence support the notion that neutrophil effector functions are not intrinsically controlled by CFTR mutations. To begin with, the fact that CFTR knockout animal models do not recapitulate neutrophilic lung inflammation as seen in CF patients itself suggests that neutrophil dysfunction in CF patients is due to one or several coinciding mechanism(s) unique to humans, besides CFTR deficiency [90]. Additional support for this idea comes from a xenograft model in which human fetal tracheal tissues were implanted in severe combined immunodeficient mice [91]. In this model, mouse neutrophils (with normal CFTR expression) were recruited to CF but not non-CF xenogratfs, emphasizing the role of the CF airway microenvironment in triggering neutrophil dysfunction. Consistently, a recent study in primary airway epithelial cultures from CF and non-CF infants showed the existence of a pro-inflammatory imbalance at steady state and upon stimulation with a viral insult in the former compared to the latter [23]. Furthermore, restoring CFTR expression in the airway epithelium of CF mice is sufficient to restore normal bacterial clearance therein, which suggests a minimal role for CFTR expression in non-epithelial cells, at least in this model [92]. Intriguingly, selective knockout of CFTR in myeloid cells in another mouse strain led to a basal inflammatory dysfunction that was further accentuated upon infection [93], suggesting that in mice, the impact of CFTR on myeloid cells (including but not limited to neutrophils) may be dependent upon the strain and conditions tested.

From a microbiological perspective, the predominance of a handful of bacterial species in CF lungs in vivo suggests that neutrophil-mediated clearance may not be completely abolished in this setting, but rather that distinct evolutionary pressures are at play that are unlikely to be solely driven by CFTR-dependent dysregulation of neutrophil phagocytosis or degranulation. Indeed, a recent study showed that the CF lung pathogen *P. aeruginosa* is resistant to neutrophil-mediated extracellular killing, a process that is CFTR-independent [94]. Furthermore, if neutrophils in CF patients were intrinsically defective due to endogenous CFTR dysfunction, one would expect evidence of chronic infection and inflammation in organs other than the lungs, which is not the case.

It is also noteworthy that in COPD and non-CF bronchiectasis patients devoid of a hereditary CFTR defect, massive neutrophil transmigration also occurs in the lungs, with subsequent release of primary granules and impaired phagocytosis reminiscent of the picture seen in CF patients [95, 96]. This suggests that a primary defect in CFTR expression is not the root cause of neutrophilic inflammation in these disease contexts. It remains possible, however, that CFTR expression may be intrinsically normal in these patients, only to be downregulated post-translationally due to high extracellular activity of NE, thus affecting neutrophil fate [97]. In CF patients, chronic disease may lead to similar adaptive changes in blood neutrophils. This could account, for example, for the observed dysfunction of Rab27a in blood neutrophils from adult CF patients, a key protein involved in tertiary and secondary granule exocytosis, coupled with the finding that significant improvement in Rab27a function in these neutrophils can be brought upon by *ex vivo* treatment with the CFTR potentiator ivacaftor [98]. Proving the existence of an intrinsic defect in neutrophils in CF patients would ultimately require well-controlled data in infants, prior to the advent of chronic disease, a feat that has not been achieved so far. These and other novel approaches and experimental designs will be necessary to further elucidate the etiology of the abnormal neutrophil effector functions that are manifest in CF lungs.

#### Immunomodulatory role of neutrophils

Since the early 1960s, significant heterogeneity among circulating and tissue neutrophils has been recognized, and this notion has gained further traction recently as evidence of divergent immunomodulatory functions by neutrophil subsets are emerging in different pathological contexts [99]. One critical example lies in tumor microenvironments, where neutrophils can display a strong immunosuppressive phenotype, promoting tumor survival [100, 101]. As immunomodulatory cells, neutrophils modulate not only their own kin but also a variety of other immune and structural cells. To do so, neutrophils use a variety of chemokines and cytokines that regulate other cells acutely and have the potential to induce chronic signaling loops that shape the long-term immune response [102, 103].

In CF, the lumen of the lung is brimming with neutrophils, while it is conspicuously devoid of T cells. The enzyme arginase-1 can be released by neutrophils, as well as M2-polarized macrophages and dendritic cells, leading to depletion of extracellular arginine, which in turn can inhibit T cell activity. Arginase-dependent T cell inhibition is common in tumor microenvironments [104] and upon infection with certain viruses [105]. In CF lungs, arginase-1 is released by neutrophils, making the airway lumen a highly inhibitory milieu for T cells [106]. In addition to the strong inhibitory role played by neutrophil-derived arginase-1, neutrophil-derived elastase can also cleave multiple critical T cell coreceptors, therefore blocking T cell activation (see references [107] and [108], and Table 1). Furthermore, programmed death ligand 1 (PD-L1), a known inhibitor of T cell function in a variety of pathologies and a major target for immunotherapy [109], was also found to be expressed in human airway neutrophils at higher levels than on their blood counterparts (in both CF and healthy subjects), but with a characteristic bimodal expression in CF. In addition, soluble PD-L1 was also detected in CF, but not healthy, airway fluid [106]. The precise role of cell-associated and soluble PD-L1 on T cell modulation in CF remains to be fully explored.

Interestingly, the impact of CF airway neutrophils on T cell function may not be solely inhibitory, since these cells were shown to increase expression of the T-activatory surface receptors major histocompatibility complex II, co-activator CD80, and prostaglandin D2 receptor CD294, further underlining their plasticity [69]. Expression of major histocompatibility complex II and CD80 is conventionally thought to be the prerogative of professional antigen-presenting cells, such as dendritic cells and macrophages, while CD294 is a marker for Th2-polarized immune cells in the context of allergy and hypersensitivity reactions [110]. The exact role of these T-activatory proteins on the surface of CF airway neutrophils has yet to be determined, although one can speculate a possible role in skewing T cell responses that may occur in spite of arginase-1 and NE-dependent inhibition. Indeed, it has been observed that CD4^+^ T cells in CF mouse models [111] and human CF airway samples and tissues [112–115] are skewed toward pro-inflammatory Th2/Th17 responses, while inhibitory T-regulatory function is inhibited.

Positive regulation of T cells by neutrophils was also suggested in early-stage human non-small cell lung cancer [116], in which tumor-associated neutrophils expressing typical antigen-presenting cell markers were able to induce T cell activation *ex vivo*. A recent study has also shown the importance of neutrophils in promoting a protective Th17 T cell response upon vaccination against tuberculosis [117]. Since Th17 cells and their product IL-17 create a positive feedback loop for neutrophil recruitment by tissues [118], neutrophil/T cell interplay may be critical to pathogenesis in CF and other relevant diseases. Rheumatoid arthritis (RA) is another example of a chronic disease in which neutrophils recruited from blood to the synovium dominate signaling loops to induce a skewed immune response [119].

#### Metabolic licensing of neutrophils

The CF lung lumen is a very peculiar microenvironment in terms of oxygen, and metabolite content. The normal lung lumen is oxygen-rich due to constant breathing activity. However, in diseased areas within the CF lung lumen, neutrophil clusters, bacterial and/or fungal colonies, and inspissated extracellular scaffolds of mucus, DNA, and actin can lead to profound oxygen depletion. Local hypoxia can in turn promote inflammation through the release of DAMPs from host epithelial cells [35, 120, 121]. Furthermore, the CF lung environment has a distinct metabolite composition, presumably as a consequence of both CFTR dysfunction and of the chronic presence of neutrophils and microbiota in the lumen. First, it has been suggested that CFTR, although functioning primarily as a chloride and bicarbonate channel [122], can also enable transmembrane flux of the redox intermediates glutathione (GSH) and thiocyanate [123–125]. CFTR is also indirectly involved in the control of neutral amino acid transport across the epithelium [126]. In addition, the CF lung lumen was shown to contain abnormal levels of nucleotides [127], glucose, and peptides [128].

The composition of the CF airway milieu drives adaptations in neutrophils, and in turn, these adaptations influence this pathological microenvironment. A telling example is that of the redox imbalance that constitutes a hallmark of CF. Local and systemic accumulation of oxidants are believed to impact CF blood neutrophils, which display lower intracellular GSH levels [69]. Meanwhile, reactive oxygen species produced by neutrophils including hypochlorous acid (bleach), a byproduct of the enzyme MPO exocytosed from primary granules at the same time as NE, can quickly and profoundly oxidize the lung microenvironment [129]. Finally, neutrophils can contribute to extracellular GSH catabolism, by expressing at their surface the GSH-metabolizing enzyme gamma-glutamyltransferase [130]. Another example is that of arginine, which neutrophils can deplete from the CF airway lumen by releasing arginase-1 [131] from their granules [132]. Consequently, low availability of arginine results in decreased nitric oxide production [133] and high levels of arginine degradation products ornithine and polyamines [134].

From an intracellular signaling standpoint, comparative studies conducted in blood and airway neutrophils collected from CF patients in vivo showed that these do not differ with regard to their levels of active, phosphorylated forms of the critical intermediate kinases Akt, c-Jun-related kinase, p38 mitogen-activated protein kinase, and p44/42 extracellular-regulated kinase or of the pro-inflammatory transcription factors, nuclear factor κB p65, and signal transducer and activator of transcription 5 [69]. However, CF airway neutrophils had increased levels of phosphorylated forms of effector proteins in the mammalian target of rapamycin pathway, a major anabolic switch. These included phosphorylated S6 ribosomal protein [69], eukaryotic initiation factor 4E, and 4E-binding protein 1 [32]. Additionally, levels of the phosphorylated cyclic AMP-response element binding protein, as well as its upstream sensors, CD114 and receptor for advanced glycation endproducts, and downstream targets, CD39 and CXCR4, which function together as another anabolic switch in cells, were found to be increased in CF airway neutrophils [32]. In aggregate, these results suggest that CF airway neutrophils are licensed by the microenvironment to become anabolic, i.e., to use resources at their disposal to survive and expand their functions.

Insights gained from the analysis of intracellular phosphorylation cascades in CF airway neutrophils were confirmed by analysis of nutrient transporter expression. Compared to their blood counterparts, CF airway neutrophils as a whole displayed high expression of the inorganic phosphate transporter PiT1 (necessary for ATP synthesis) and of the glucose transporter Glut1, coinciding with increased glucose uptake [135]. Subset analysis of CF airway neutrophils showed that the degranulation of primary granules typical of the A2 subset was associated with higher expression of Glut1 and PiT1, and of the other inorganic phosphate transporter PiT2. Expression of CD98, a shared subunit of multiple amino acid transporters, did not differ in CF airway compared to blood neutrophils [32], but that of ASCT2, a neutral amino acid transporter, was highly upregulated in the A2 subset [135]. It is likely that this metabolic surge leads to de novo transcription and protein production in CF airway neutrophils, since neutrophils recruited to other pathological environments, e.g., the synovium of RA patients, have shown an ability to increase mRNA output [49, 136, 137].

The combined massive and sustained recruitment of neutrophils from blood into CF lungs (presumably leading to an increased neutrophil production in the BM), and increased metabolic activity of these neutrophils once they have reached the CF airway lumen are expected to impact lung tissue function and systemic metabolism in patients. Indeed, the severity of CF lung inflammation has been correlated not only to a decreased lung function [138] but also to decreased body mass index [61], decreased heart function [139], and cardiovascular complications [140]. Generally, the establishment of a complex microenvironment involving not only the chronic and massive presence of neutrophils but also large populations of bacteria (and/or fungi) in the airway lumen may increase the metabolic share taken by the lung, at the expense of other organs [141].

The relationship between inflammation, high cellular turnover, and increased systemic energy expenditure is not confined to CF, but rather is a common feature of an array of chronic human diseases. For example, high body mass index at late cancer stages predicts a higher survival rate [142]. Understanding the mechanism underlying the anabolic switch in CF airway neutrophils and the interplay between the different actors within the CF lung microenvironment could help identify treatments impacting not only lung disease but also the overall metabolic balance in patients.

#### The CF airway microenvironment

The existence of discrete microenvironments within the human body is not a new concept. This concept is exemplified by the gut mucosa, featuring fine-tuned interplay between the resident flora in the lumen and the immune cells in the lamina propria, with the epithelium as an interface. While the gut mucosa represents a normal microenvironment, it can also become imbalanced in the context of inflammatory bowel disease. Other pathological conditions can lead to the formation of microenvironments in organs that do not normally harbor a significant, stable population of inflammatory cells and/or associated microbiota. CF, RA, and several forms of cancer serve as examples of such pathological microenvironments featuring a dominant neutrophilic component.

A key element in the formation of pathological microenvironments is the establishment of tolerance and cooperation between the different players, enabling an acute process to become chronicized. For instance, inflammatory bowel disease is characterized by a massive neutrophil infiltration in the gut [143, 144] and as the disease progresses, neutrophils orchestrate with the gut epithelium the advent of a chronic state. Consequently, adaptive responses are dampened [145], and neutrophils and bacteria coexist in concentric luminal structures termed “casts” [146]. A similar scenario unfolds in CF, where neutrophils interact with the airway epithelium and opportunistic bacteria such as *P. aeruginosa* and *S. aureus* to establish multi-decade colonies, featuring minimal involvement of adaptive immune cells, and structures in the lumen where bacteria and neutrophils coexist [147].

**Fig. 2:**
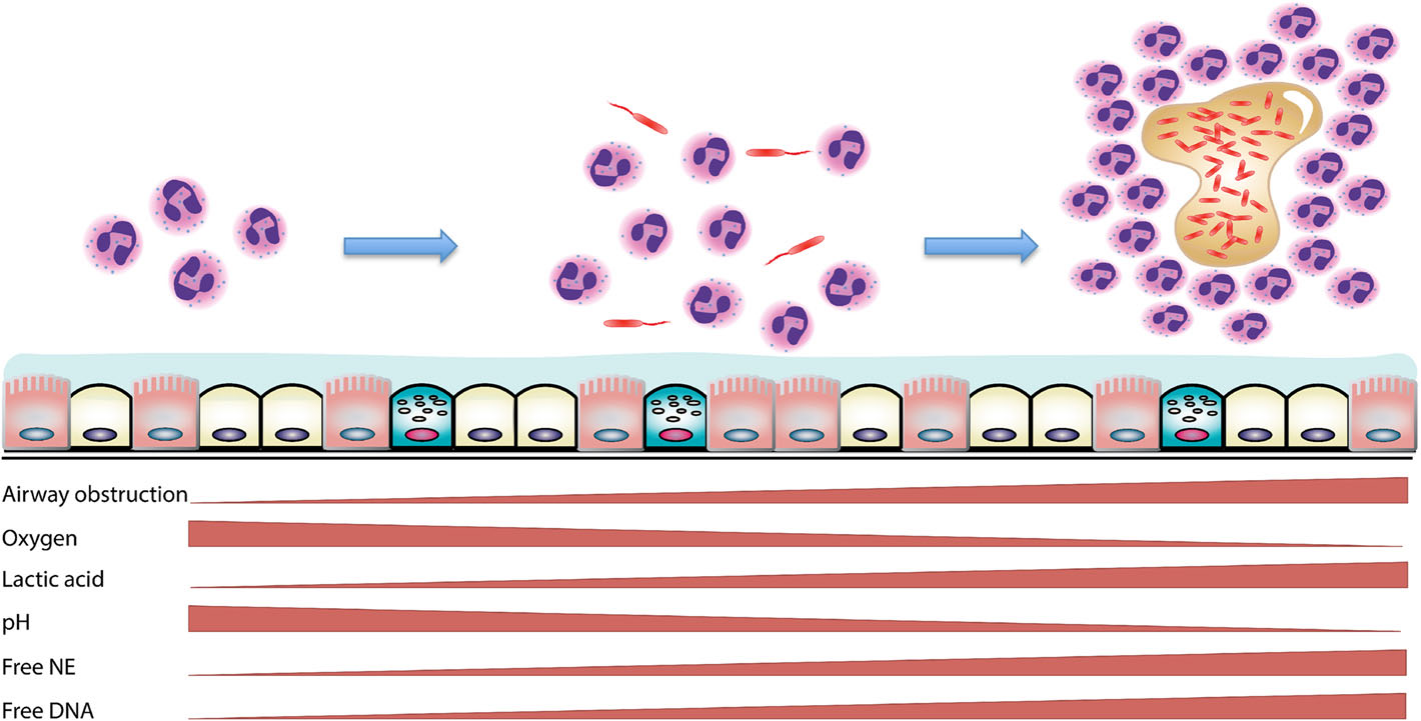
Development and evolution of a pathological microenvironment in CF airways. From birth to adulthood, CF patients undergo stepwise changes to their airway microenvironment. In the first phase (*left*), neutrophil start to accumulate in the lumen in response to cues from the underlying epithelium, prior to the advent of chronic infection. The second phase (*middle*) features the stable coexistence of an even more substantial population of luminal neutrophils with planktonic bacteria (*in red*, drawn with flagellum to exemplify *P. aeruginosa*, the gram-negative pathogen most commonly found in CF patients). In the third phase (*right*), bacteria switch to a resistant and generally avirulent and auxotrophic mode of existence, encased in an extracellular molecular scaffold (mucoid or biofilm forms, *in yellow*), with a very large population of neutrophils organized as a cast around them. Shown under the epithelium are the critical environmental conditions that change during the formation of this pathological microenvironment, including the degree of airway obstruction (increasing), and luminal levels of oxygen (decreasing as neutrophil activate their reactive oxygen species burst) and lactic acid (increasing with neutrophil glycolytic activity), pH (decreasing as lactic acid accumulates), as well the burden of free NE and DNA (both correlated positively with neutrophil presence)

Recent studies suggest that the formation of pathological microenvironments featuring substantial relocation of hematopoietic cells within a peripheral organ can impact other distal organs besides the BM, where hematopoiesis takes place. For example, Masri et al. [148] have shown that establishment of a lung tumor mass can lead to re-tuning of the liver circadian clock and reprogramming of its nutrient output in order to support the metabolic requirements of the remote lung tumor. Whether the establishment of a pathological microenvironment in the CF lung does not only involve complex local coordination but also impact other organs in the body (notably the liver and gut, in order to respond to its metabolic demands) remains to be established. Taken together, evidence from in vivo and in vitro studies suggest that, at a minimum, the airway epithelium, recruited neutrophils, and bacteria present in the CF microenvironment coevolve over time to enable a somewhat peaceful, albeit tissue-damaging, coexistence, as illustrated in Fig. 2.

#### Treatment opportunities

The development of drugs aimed to treat CF has mainly focused on fluidifying secretions, regulating microbial burden and, more recently, rescuing mutant CFTR function. The latter approach has paid substantial dividends with the drug ivacaftor for CFTR gating mutants such as G551D. However, the downside of this approach is that it is mutation-specific and benefits only a small percentage of CF patients [149]. So far, only little attention has been paid to the regulation of neutrophil function, since the long-held view has been that these cells die quickly upon migration to CF lungs. Data discussed in this review clearly contradict this view and open new avenues for neutrophil-focused therapies in CF.

Conventional anti-inflammatory drugs, including ibuprofen and prednisone, have shown beneficial, albeit marginal, effects by slowing down CF disease progression [150, 151]. However, prednisone treatment of CF patients is not common due to important side effects on growth [152]. More recent efforts focused on drugs designed to inhibit neutrophil recruitment to the CF lung, such as BIIL 284, a LTB_4_ receptor antagonist [153], and SB 656933, a CXCR2 antagonist [154]. Both drugs led to an increase in inflammatory signaling (increased frequency of exacerbations for the former, and increased circulating inflammatory mediators for the latter), suggesting that inhibiting neutrophil recruitment to the CF lung may prove detrimental for patients [155]. In addition to inhibiting neutrophil recruitment into the lung lumen, BIIL 284 was also found to promote apoptosis of neutrophils that had transmigrated [156]. Arguably, focus should now be put on the development of drugs directed toward regulating neutrophil function or aiming to orchestrate their chemotaxis to the lung to attain normal homeostatic levels, rather than blocking their recruitment, which could lead to detrimental, sub-normal levels of these cells within the lung lumen.

Since NE activity is elevated in CF patients and correlates with disease progression, development of NE inhibitors has been of prime interest [157, 158]. Unfortunately, due to the high amount of NE present in CF airways, its broad range of substrates, and its compartmentalization as both a free-floating, mucus-associated, and membrane-bound enzyme [55], the design of inhibitors and modes of administration have to be significantly improved to attain therapeutic efficacy [159]. In a recently introduced approach, Forde et al. [160] leveraged NE activity in diseased airways to process a synthetic pro-drug, giving rise to a fully active anti-infectious small peptide. A similar approach could be applied to design NE-activated immunomodulatory drugs. Examples of relevant immunomodulatory drugs includes agents able to (i) target the organizing stage of neutrophil swarming preceding their transepithelial migration, which may reduce, as opposed to fully abrogate their recruitment to the lung; (ii) manipulate the metabolism and/or functional fate of neutrophils, to promote phagocytosis while reducing NETosis and degranulation/reprogramming; and (iii) regulate the lifespan of airway neutrophils or interfere with other factors enabling the establishment of a pathological, neutrophil-driven microenvironment in CF lungs.

## Conclusions

**Fig. 3:**
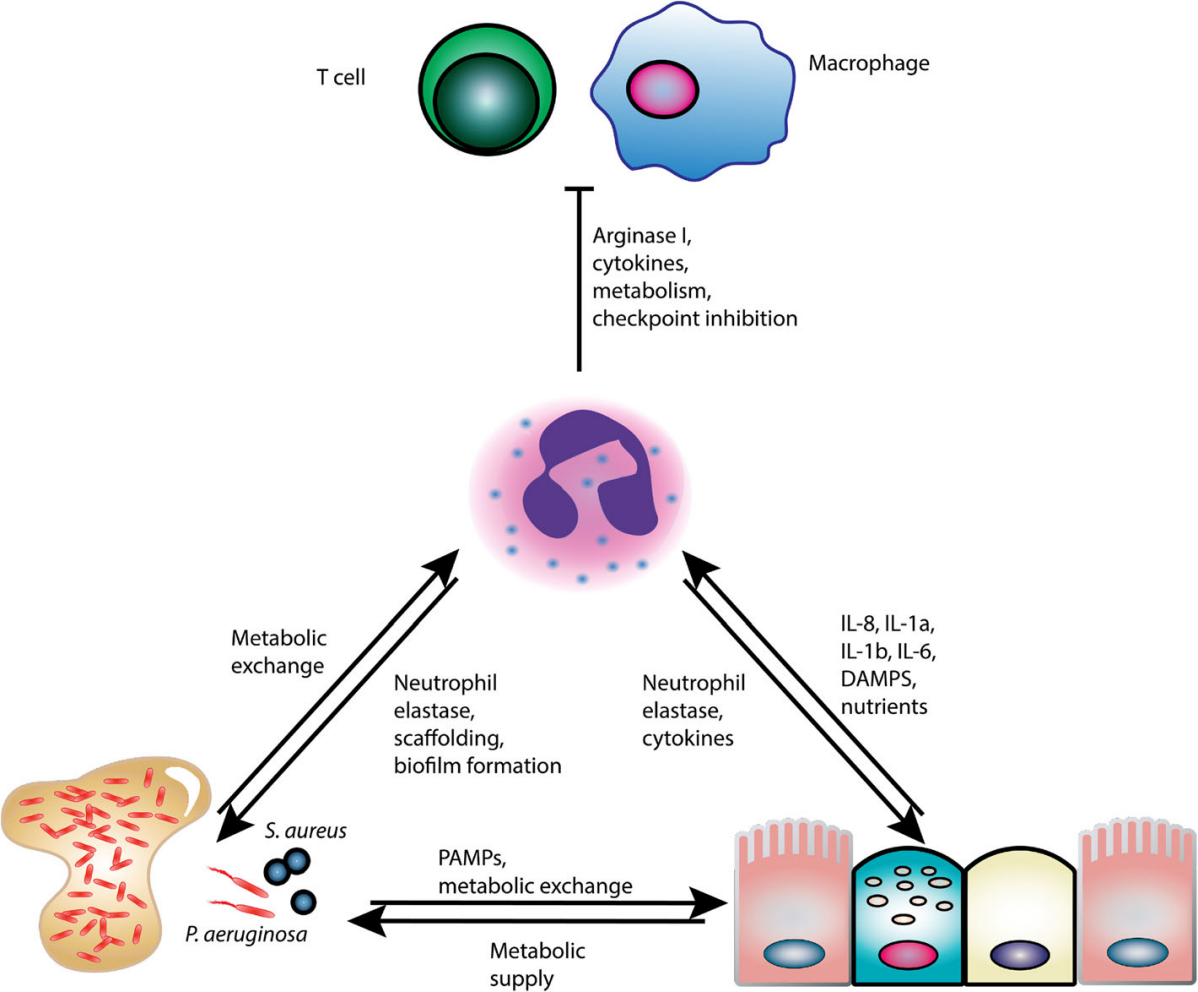
Neutrophils as protagonists among contributors to CF airway disease. Neutrophils (*center*) establish metabolic and signaling ties with lung epithelial cells (*bottom right*) and resident planktonic and mucoid/biofilm bacteria (*bottom left*), while exerting primarily inhibitory control over other immune cells, including T cells and macrophages (*top*). The result of these complex relationships is the formation of a relatively stable pathological microenvironment within CF airways

In the last decade, monumental progress has been made to understand the processes characterizing the peculiar situation of CF lung disease. Despite extensive work, there is much more to be explored regarding neutrophil functions and plasticity, and their ability to occupy a central place in the development of a pathological microenvironment in CF lungs (Fig. 3).

**Fig. 4:**
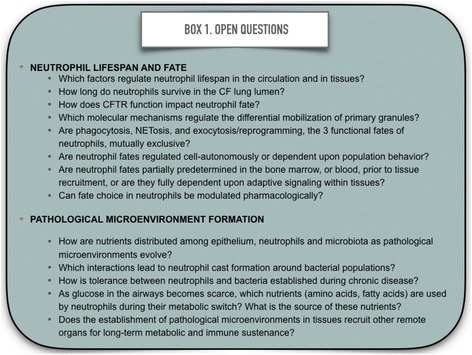
Open questions related to human neutrophil fate and their role in the formation of pathological microenvironments

Neutrophils currently enjoy renewed interest from basic and clinical researchers, as emerging evidence supports the idea that mechanisms of metabolic and functional plasticity described here are not confined to CF. Therefore, a better understanding of molecular mechanisms underlying neutrophil plasticity and neutrophil-epithelium-microbial partnership should help identify novel targets for treatments aiming to normalize pathological microenvironment development in CF, and similar neutrophil-driven diseases such as COPD, RA, and certain forms of cancer. Expanding our knowledge in terms of crosstalk between metabolic switching, interconnecting pathways and effector functions in neutrophils will be of high value for innumerable reasons. To close this review, we invite readers to consult Fig. 4, which lists several of the open questions pertaining to neutrophil plasticity and function that will need to be addressed in the near future.

## Abbreviations

BALF: Bronchoalveolar lavage fluid
BM: Bone marrow
CF: Cystic fibrosis
CFTR: Cystic fibrosis transmembrane conductance regulator
COPD: Chronic obstructive pulmonary disease
DAMPs: Damage-associated molecular patterns
GSH: Glutathione
HIF1α: Hypoxia-inducible factor 1α
IL-8: Interleukin-8
MMP9: Matrix metalloproteinase 9
MPO: Myeloperoxidase
NE: Neutrophil elastase
PAMPs: Pathogen-associated molecular patterns
PAR: Protease-activated receptor
PD-L1: Programmed death ligand 1
RA: Rheumatoid arthritis
TIMP: Tissue inhibitor of metalloproteinase
TLR: Toll-like receptor
